# Observation of Quantum Size Effect from Silicon Nanowall

**DOI:** 10.1186/s11671-016-1743-8

**Published:** 2016-11-29

**Authors:** Daiji Kanematsu, Shuhei Yoshiba, Masakazu Hirai, Akira Terakawa, Makoto Tanaka, Yukimi Ichikawa, Shinsuke Miyajima, Makoto Konagai

**Affiliations:** 1 Panasonic Corporation, Kadoma, Osaka 571-8686 Japan; 2 Japan Science and Technology Agency, Koriyama, Fukushima 963-0298 Japan; 3 Tokyo Institute of Technology, Meguro, Tokyo, 152-8552 Japan; 4 Tokyo City University, Setagaya, Tokyo, 158-0082 Japan

**Keywords:** Silicon nanowall, Quantum size effect, Ultraviolet photoelectron spectroscopy, Cathode luminescence

## Abstract

We developed a fabrication technique of very thin silicon nanowall structures. The minimum width of the fabricated silicon nanowall structures was about 3 nm. This thinnest region of the silicon nanowall structures was investigated by using cathode luminescence and ultraviolet photoelectron spectroscopy (UPS). The UPS measurements revealed that the density of states (DOS) of the thinnest region showed a stepwise shape which is completely different from that of the bulk Si. Theoretical analysis clearly demonstrated that this change of the DOS shape was due to the quantum size effect.

## Background

Multi-junction solar cells consisting of materials with different band gaps are one of the options to overcome the conversion efficiency limit of single junction solar cells [1]. Crystalline silicon (Si) is the most promising material for the bottom cell of a tandem solar cell. Recently, a material for the top cell has been widely studied [2, 3]. Si nanowire and nanowall are one of the options for the top cell material. The band gaps of Si nanowire and nanowall can be varied by changing their diameter or width owing to the quantum size effect [4], and there is the potential for high efficiency all-Si tandem solar cells. In a previous research, Si nanowires were mainly used for light-trapping structure of Si-based solar cells. In this case, the size of Si nanowires was micrometer or submicrometer range which corresponds to the wavelength of visible and infrared light [5–10]. In order to apply nanostructured Si for the top cell of all-Si tandem solar cells, it is important to reduce the size to less than 5 nm [11] to utilize quantum size effect. Therefore, techniques to fabricate extremely thin Si nanowire or nanowall are important to realize all-Si tandem solar cells.

Fabrication processes of nanostructured Si (Si nanowire or Si nanowall) are roughly divided into two types: top-down and bottom-up, i.e., etching of bulk Si [12–16] and growing Si nanowire on a substrate [17]. The advantage of the top-down process is the easy control of the direction of nanostructured Si. The starting material of this method is a Si wafer; therefore, material quality is also high enough. The typical top-down process consists of a mask patterning and anisotropic etching. The arrangement of nanostructured Si can be controlled by mask patterning. By the combination of mask patterning, e.g., nanoimprint and photolithography, and anisotropic etching, e.g., metal-assisted chemical etching (MACE) [18–20] and reactive ion etching (RIE), various processes are selectable. We have developed a device integration process of Si nanowire with a diameter of 30 nm using silica nanoparticle dispersion and MACE [21], and confirmed the photovoltaic power generation of the axial-junction Si nanowire solar cell [22]. However, the diameter of the Si nanowires was not thin enough to utilize the quantum size effect.

In this work, we succeeded to fabricate very thin Si nanowall by the combination of an etching process and a slimming process using thermal oxidation. The minimum width of the Si nanowall was 3 nm. We also investigate to confirm the quantum size effect of the Si nanowall. Si nanowall confines the carriers in one dimension; therefore, a smaller size is required to utilize the quantum size effect than Si nanowire. This is one of the disadvantages of Si nanowall; however, the Si nanowall is much stronger than Si nanowire from the viewpoint of mechanical strength. In addition, the light absorption of Si nanowall is greater than that of Si nanowire [23]. Therefore, it is important to confirm the quantum size effect of Si nanowall. In previous works, photoluminescence (PL) and scanning tunneling spectroscopy (STS) were used for confirming the quantum size effect of nanostructured Si. The PL method can measure the band gap and has been used for analysis of nanodot [24] and nanoporous structures [25]. The PL measurement includes undesirable signals such as signals from interface defects and requires high density of nanostructured Si to detect signals related to the quantum size effect. The STS method can measure the local density of states (DOS) and has been used for the analysis of single Si nanowire [26]. However, it requires an atomically flat measurement surface and is difficult to measure Si nanowire and nanowall vertical to the substrate. Therefore, we investigated our Si nanowall by using cathode luminescence (CL) and ultraviolet photoelectron spectroscopy (UPS).

## Methods

**Fig. 1:**
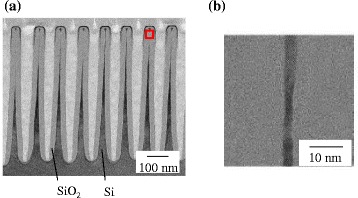
Cross-sectional TEM images of Si nanowall after thermal oxidation **a** whole image **b** magnified image of the thinnest region. A square in Fig. 1(**a**) shows the magnified area

Si nanowall was prepared by etching process using photolithography and RIE. A line and space resist pattern with a half-pitch of 55 nm was formed on a p-type single-crystalline Si wafer, and it was etched into Si nanowall. This Si nanowall has a tapered shape, and the width varied along the height direction due to the side etching during the RIE process. The width of the tips was about 20 nm. This tapered Si nanowall was slimmed by thermal oxidation. Figure 1 (a) shows the cross-sectional transmission electron microscope (TEM) images of a slimmed Si nanowall. A SiO_2_ layer covered the thin Si cores. The slimmed Si nanowall also has a tapered shape because of the initial tapered shape. The thinnest region was located at slightly below the tips since the oxidation of the tips was limited by internal stress induced in the oxide layer [27]. As shown in Fig. 1 (b), an untapered region was formed in the thinnest region of the Si nanowall. The width of this region is thin enough for the quantum size effect. Therefore, we investigate this sample by using CL and UPS in order to confirm the quantum size effect.

## Results and discussions

**Fig. 2:**
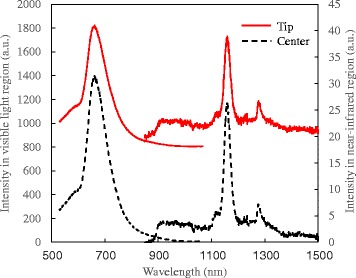
CL spectra of Si nanowall at 37 K. Tip and center of Si nanowall were irradiated by electron beam

Figure 2 shows the results of the CL measurements. Electron beam with an acceleration voltage of 20 kV is irradiated to the tip and center of the Si nanowall from the cross-sectional surface. The measurement temperature was 37 K. Similar spectra were obtained from the tip and center of the Si nanowall. The peaks at 1130 and 1170 nm correspond to the phonon-assisted band-to-band emission of Si [28]. This can be interpreted as follows. The injected electrons near the tip immediately diffused toward the bottom of the Si nanowall, and the emission occurred in all regions of the Si nanowall if the electron beam was irradiated only to the tip. In this situation, the emission from the thick region was superimposed on emission from the tip [29]. The broad peak at around 660 nm observed from both the tip and the center was assigned to the emission related to defects in the oxide layer [30, 31]. Comparing the two spectra, we could not find clear difference. This means that it is difficult to detect the quantum size effect by using the CL measurements. The emission signal from the thinnest region of the Si nanowall is very weak since the volume of the thinnest region is very small. In this case, the emission signal from the thick region caused by the electron diffusion obscures the signal from the thinnest region. Therefore, signals from the thick region and the oxide layer have to be excluded to detect the signal from the thinnest region.

In order to confirm the quantum size effect, we also analyzed the slimmed Si nanowall by UPS. A helium discharge tube was used as the light source and UV light with energy of 40.8 eV was irradiated to the tips of the Si nanowall. The kinetic energy of the photoelectrons emitted from a sample is influenced by the work function and the binding energy. Therefore, an UPS spectrum reflects the density of states in the valence band [32]. The most important advantage of UPS is high surface sensitivity. The maximum kinetic energy of electrons in this measurement is 40.8 eV, which corresponds to the mean free path of electrons less than 1 nm [33]. This indicates that the UPS can only measure the DOS of the surface of the sample. Therefore, we can selectively detect the UPS signal of the tips of the Si nanowall if we can prepare the sample in which the tips are located at the surface.

**Fig. 3:**
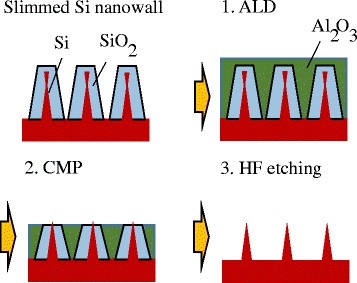
Sample fabrication process for UPS measurement

**Fig. 4:**
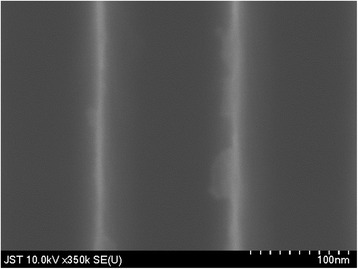
Overhead SEM image of bared Si nanowalls after HF etching. The Si nanowalls have a tapered shape as shown in Fig. 1. Sharp and clear regions thinner than 10 nm are tips of Si nanowall. The blurred region around the tips corresponds to the bottom region of the nanowall

Figure 3 shows the sample fabrication process. The spaces in the Si nanowall were filled with Al_2_O_3_ deposited by using atomic layer deposition (ALD). The tips of the Si nanowall were bared by chemical mechanical polishing (CMP). Then, the SiO_2_ and Al_2_O_3_ layers were removed by 5% HF etching. Figure 4 shows the scanning electron microscope (SEM) image of the bared Si nanowall. It was confirmed that Si nanowall was standing independently, and the width of the tip is 3 nm. This width corresponds to a theoretical band gap expansion of about 0.2 eV [11]. Just after the HF etching, the surface of Si nanowall was terminated by hydrogen; hence, the quantum size effect can be expected to be confirmed.

**Fig. 5:**
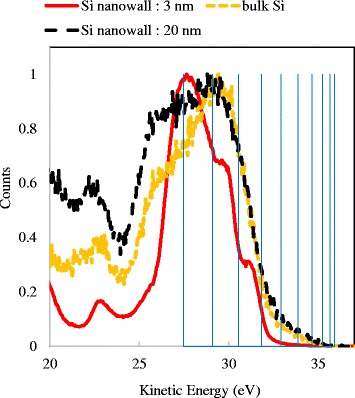
UPS measurement results of Si nanowall with different widths: 3 and 20 nm and bulk. The incidence angle was 50°. The counts values were normalized at the maximum values

Figure 5 shows the results of the UPS measurements. We prepared three types of samples, namely slimmed Si nanowall with a 3-nm width, an unslimmed one with a 20-nm width, and bulk Si. The value of the vertical axis, counts, reflects the DOS. The onset of the increase in the counts near a kinetic energy of 36 eV corresponds to the upper end of the valence band *E*_V_. However, the shift of *E*_V_ edge was not observed. This is probably due to the tapered shape of Si nanowall as shown in Fig. 2. The surface sensitivity of UPS was less than 1 nm. However, the incident ray was at an angle of 50° to the Si nanowall. The thick region of the Si nanowall below the tips was irradiated with the ultraviolet ray from the sidewall as well as the tips and photoelectrons were emitted. In this case, UPS signals from the tips and the thicker region are simultaneously detected and the shift of *E*_V_ edge was not observed.

**Fig. 6:**
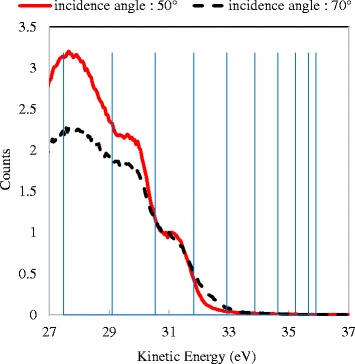
UPS measurement results of the Si nanowall with a width of 3 nm at different UV irradiation angles: 50° and 70°. The counts values were normalized at the step with *n* value of 7

However, a characteristic DOS structure of 3-nm-width Si nanowall was confirmed. The DOS structures of the 20-nm-width Si nanowall were similar to that of bulk Si, whereas the DOS structure changed into a stepwise shape when the thickness of Si nanowall was 3 nm. We also investigated the change of the DOS structure in order to clarify the quantum size effect. In the case of a quantum well, it is known that the DOS increases at the quantum level stepwise [34]. The quantum level can be calculated by

1 $$ {\varepsilon}_n=\frac{{\left(\hslash \pi n\right)}^2}{2{m}^{*}{L}^2} $$

where *ε*_*n*_ is the quantum level, *ħ* is the reduced Planck constant, m* is the effective mass of the hole, *L* is the width of quantum well, and *n* is the quantum number. In the case of the valence band, the quantum levels appear at *ε*_*n*_ below *E*_V_. The calculated quantum levels were added in Fig. 5. In this calculation, the effective mass of bulk Si and the width of Si nanowall were used for m* and *L*, respectively. The Fermi level *E*_F_ of electrode was 36.45 eV which coincided with the *E*_F_ of Si nanowall. The measured sample was p-type Si, so the energy level of *E*_V_ exists between 36.45 and 35.89 eV. We assumed the *E*_V_ to be 36.0 eV which is the onset of the increase in the counts. As shown in Fig. 5, the onset of each step of the DOS corresponds to the quantum level with *n* = 7, 8, and 9. Strictly speaking, the calculated quantum levels were slightly smaller than the onset levels. Considering the relationship between the *E*_V_ and *E*_F_, the *E*_V_ of 36.0 eV may be slightly underestimated. The quantum levels with small *n* values were not observed. In the region with small *n* values, intervals of the quantum levels become small. The quantum level is varied by the thickness of the Si nanowall; therefore, near the band edge, it was buried in the signal from the thick region because of the tapered shape. Figure 6 shows the measurement result with UV incidence angles of 50° and 70°. By irradiating the sample at a shallow angle, the sensitivity to the tips can be high. When the incidence angle is 70°, lower peak corresponding to the *n* value of 6 was observed by comparing with the measurement at an angle of 50°. Although the DOS structure corresponds to the small *n* values was not observed, the band gap widening can be estimated from the quantum levels. The first quantum level means an energy shift of *E*_V_ and it was calculated as 0.085 eV. Thereby, the band gap widening can be estimated to be about 0.2 eV, along with the conduction band shift. This value corresponds to the theoretical band gap widening of the quantum well with a width of 3 nm.

## Conclusions

We investigated properties of an extremely thin Si nanowall in which the width of the thinnest region was 3 nm. We found that CL measurement is not suitable to detect the quantum size effect due to the undesirable luminescence caused by the diffusion of injected electrons and the influence of the oxide layer. We also fabricated a slimmed Si nanowall without the oxide layer and measured it by UPS. When the width of Si nanowall was 3 nm, the change of the DOS structure in the valence band was observed. According to the comparison between the experimental DOS structure and the theoretical quantum levels, we concluded that this change in the DOS is caused by the quantum size effect.

## References

[1] Dimroth F, Grave M, Beutel P, Fiedeler U, Karcher C, Tibbits TND, Oliva E, Siefer S, Schachtner M, Wekkeli A, Bett AW, Krause R, Piccin M, Blanc N, Drazek C, Guiot E, Ghyselen B, Salvetat T, Tauzin A, Signamarcheix T, Dobrich A, Hannappel T, Schwarzburg K (2014). Wafer bonded four-junction GaInP/GaAs//GaInAsP/GaInAs concentrator solar cells with 44.7% efficiency. Prog Photovolt.

[2] Löper P, Moon SJ, Nicolas SM, Niesen B, Ledinsky M, Nicolay S, Bailat J, Yum JH, Wolf SD, Balli C (2015). Organic–inorganic halide perovskite/crystalline silicon four-terminal tandem solar cells. Phys Chem Chem Phys.

[3] Bertness KA, Kurtz SR, Friedman DJ, Kibbler AE, Kramer C, Olson JM (1994). 29.5%‐efficient GaInP/GaAs tandem solar cells. Appl Phys Lett.

[4] Priolo F, Gregorkiewicz T, Galli M, Krauss TF (2014). Silicon nanostructures for photonics and photovoltaics. Nature Nanotech.

[5] Dossou KB, Botten LC, Asatryan AA, Sturmberg BCP, Byrne MA, Poulton CG, McPhedran RC, Sterke CM (2012). Modal formulation for diffraction by absorbing photonic crystal slabs. J Opt Soc Am A.

[6] Zhang X, Pinion CW, Christesen JD, Flynn CJ, Celano TA, Cahoon JF (2013). Horizontal silicon nanowires with radial p−n junctions: a platform for unconventional solar cells. J Phys Chem Lett.

[7] Hu L, Chen G (2007). Analysis of optical absorption in silicon nanowire arrays for photovoltaic applications. Nano Lett.

[8] Garnett E, Yang P (2010). Light trapping in silicon nanowire solar cells. Nano Lett.

[9] Wang J, Li Z, Singh N, Lee S (2011). Highly-ordered vertical Si nanowire/nanowall decorated solar cells. Opt Express.

[10] Sturmberg BCP, Dossou KB, Botten LC, Asatryan AA, Poulton CG, Sterke CM, McPhedran RC (2011). Modal analysis of enhanced absorption in silicon nanowire arrays. Opt Express.

[11] Kurokawa Y, Kato S, Watanabe Y, Yamada A, Konagai M, Ohta Y, Niwa Y, Hirota M (2012). Numerical approach to the investigation of performance of silicon nanowire solar cells embedded in a SiO_2_ matrix. Jpn J Appl Phys.

[12] Huang Z, Geyer N, Werner P, Boor J, Gösele U (2011). Metal-assisted chemical etching of silicon: a review. Adv Mater.

[13] Peng K, Lu A, Zhang R, Lee ST (2008). Motility of metal nanoparticles in silicon and induced anisotropic silicon etching. Adv Funct Mater.

[14] Li X, Xiao Y, Bang JH, Lausch D, Meyer S, Miclea PT, Jung JY, Schweizer SL, Lee JH, Wehrspohn RB (2013). Upgraded silicon nanowires by metal-assisted etching of metallurgical silicon: a new route to nanostructured solar-grade Silicon. Adv Mater.

[15] Chang SW, Chuang VP, Boles ST, Ross CA, Thompson CV (2009). Densely packed arrays of ultra-high-aspect-ratio silicon nanowires fabricated using block-copolymer lithography and metal-assisted etching. Adv Funct Mater.

[16] Chang SW, Chuang VP, Boles ST, Thompson CV (2010). Metal-catalyzed etching of vertically aligned polysilicon and amorphous silicon nanowire arrays by etching direction confinement. Adv Funct Mater.

[17] Yan HF, Xing YJ, Hang QL, Yu DP, Wang YP, Xu J, Xi ZH, Feng SQ (2000). Growth of amorphous silicon nanowires via a solid–liquid–solid mechanism. Chem Phys Lett.

[18] Tsujino K, Matsumura M (2005). Helical nanoholes bored in silicon by wet chemical etching using platinum nanoparticles as catalyst. Electrochem Solid-State Lett.

[19] Zhang ML, Peng KQ, Fan X, Jie JS, Zhang RQ, Lee ST, Wong NB (2008). Preparation of large-area uniform silicon nanowires arrays through metal-assisted chemical etching. J Phys Chem C.

[20] Hildreth OJ, Lin W, Wong CP (2009). Effect of catalyst shape and etchant composition on etching direction in metal-assisted chemical etching of silicon to fabricate 3D nanostructures. ACS Nano.

[21] Kato S, Watanabe Y, Kurokawa Y, Yamada A, Ohta Y, Niwa Y, Hirota M (2012). Metal-assisted chemical etching using silica nanoparticle for the fabrication of a silicon nanowire array. Jpn J Appl Phys.

[22] Kanematsu D, Yata S, Terakawa A, Tanaka M, Konagai M (2015). Photovoltaic properties of axial-junction silicon nanowire solar cells with integrated arrays. Jpn J Appl Phys.

[23] Kanematsu D, Yata S, Terakawa A, Tanaka M, Konagai M (2015). Effective light trapping by modulated quantum structures for Si nanowire/wall solar cells. Jpn J Appl Phys.

[24] Kurokawa Y, Tomita S, Miyajima S, Yamada A, Konagai M (2007). Photoluminescence from silicon quantum dots in Si quantum dots/amorphous SiC superlattice. Jpn J Appl Phys.

[25] Gelloz B, Loni A, Canham L, Koshida N (2012). Luminescence of mesoporous silicon powders treated by high-pressure water vapor annealing. Nanoscale Res Lett.

[26] Ma DDD, Lee CS, Au FCK, Tong SY, Lee ST (2003). Small-diameter silicon nanowire surfaces. Science.

[27] Liu HI, Biegelsen DK, Johnson NM, Ponce FA, Pease RFW (1993). Self-limiting oxidation of Si nanowires. J Vac Sci Technol B.

[28] Davies G (1989). The optical properties of luminescence centres in silicon. Phys Rep.

[29] Kanaya K, Okayama S (1972). Penetration and energy-loss theory of electrons in solid targets. J Phys D.

[30] Yoshikawa M, Matsuda K, Yamaguchi Y, Matsunobe T, Nagasawa Y, Fujino H, Yamane T (2002). Characterization of silicon dioxide film by high spatial resolution cathodoluminescence spectroscopy. J Appl Phys.

[31] Watanabe M, Juodkazis S, Sun HB, Matsuo S, Misawa H (1999). Luminescence and defect formation by visible and near-infrared irradiation of vitreous silica. Phys Rev B.

[32] Rowe JE, Ibach H (1974). Surface and bulk contributions to ultraviolet photoemission spectra of silicon. Phys Rev Lett.

[33] Pi TW, Hong IH, Cheng CP, Wertheim GK (2000). Surface photoemission from Si(100) and inelastic electron mean-free-path in silicon. J Electron Spectrosc Relat Phenom.

[34] Suresh S (2013). Semiconductor nanomaterials, methods and applications. Rev Nanosci. Nanotechnol..

